# Heme Catabolic Pathway in Inflammation and Immune Disorders

**DOI:** 10.3389/fphar.2019.00825

**Published:** 2019-07-24

**Authors:** Bing Wu, Yanwei Wu, Wei Tang

**Affiliations:** ^1^Laboratory of Immunopharmacology, Shanghai Institute of Materia Medica, Chinese Academy of Sciences, Shanghai, China; ^2^School of Pharmacy, University of Chinese Academy of Sciences, Beijing, China

**Keywords:** heme, heme oxygenase, carbon monoxide, biliverdin, inflammation, immune disorders

## Abstract

In recent years, the heme catabolic pathway is considered to play an important regulatory role in cell protection, apoptosis, inflammation, and other physiological and pathological processes. An appropriate amount of heme forms the basic elements of various life activities, while when released in large quantities, it can induce toxicity by mediating oxidative stress and inflammation. Heme oxygenase (HO) -1 can catabolize free heme into carbon monoxide (CO), ferrous iron, and biliverdin (BV)/bilirubin (BR). The diverse functions of these metabolites in immune systems are fascinating. Decades work shows that administration of degradation products of heme such as CO and BV/BR exerts protective activities in systemic lupus erythematosus (SLE), rheumatoid arthritis (RA), multiple sclerosis (MS) and other immune disorders. This review elaborates the molecular and biochemical characterization of heme catabolic pathway, discusses the signal transduction and immunomodulatory mechanism in inflammation and summarizes the promising therapeutic strategies based on this pathway in inflammatory and immune disorders.

## Introduction

**Figure 1:**
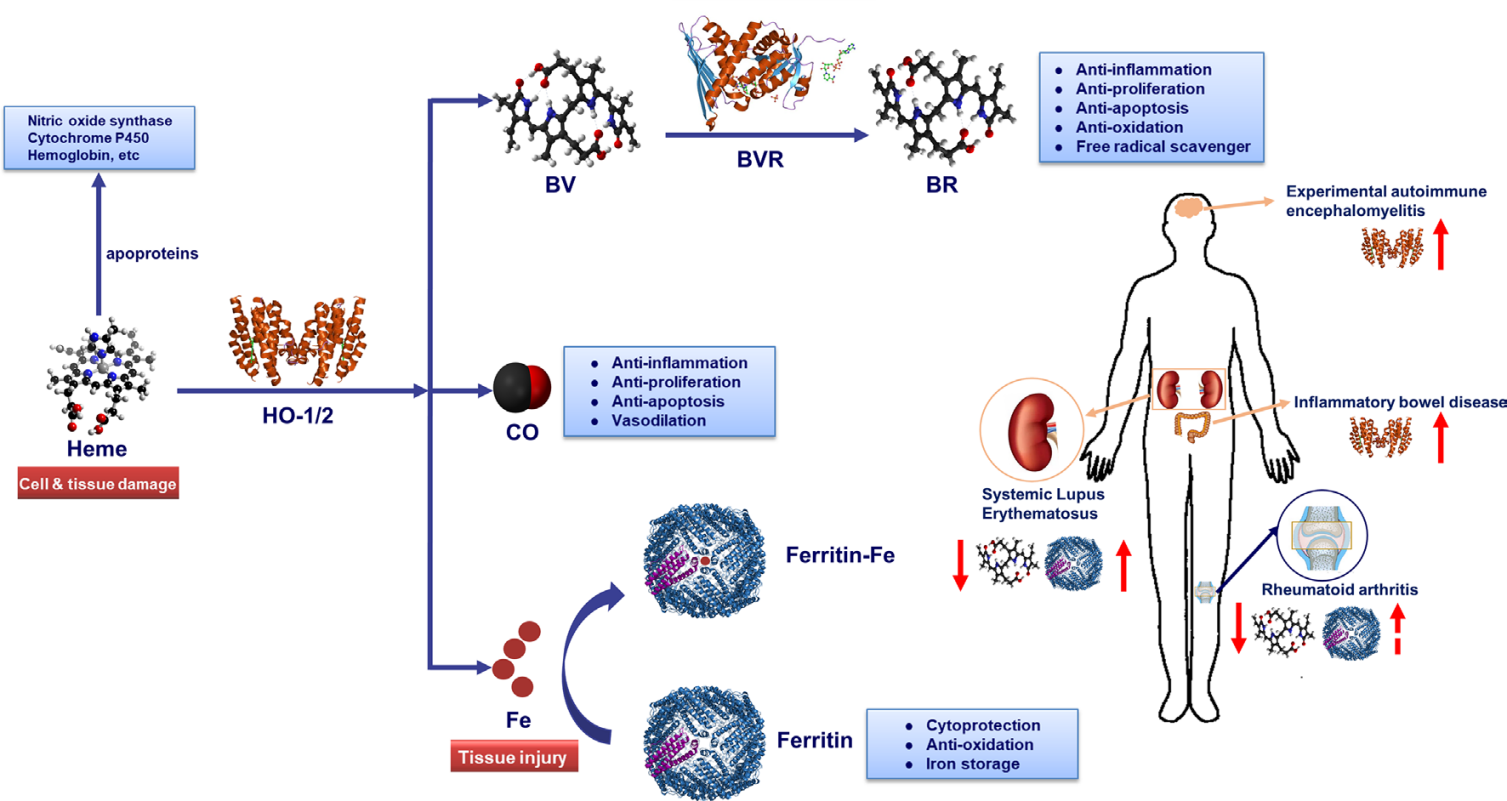
Heme metabolic pathway. Heme oxygenase (HO) catabolizes free heme into biliverdin (BV), carbon monoxide (CO) and Fe^2+^. Biliverdin (BV) is transformed into bilirubin (BR) by biliverdin reductase (BVR) enzyme. Fe^2+^ can be bound by the iron storage protein ferritin. The heme molecule provides a multitude of crucial biological functions in the form of various apo-heme proteins like hemoglobin, nitric oxide synthase, and cytochromes. HO-1-derived metabolite CO could exert anti-inflammatory, anti-proliferation, anti-apoptosis and vasodilation effects in immune system. Similarly, BR could play a key role in anti-inflammation, anti-proliferation, anti-apoptosis, anti-oxidation and free radical scavenger. Additionally, iron-induced ferritin could play a cytoprotective, anti-oxidative and iron storage effect. Notably, HO-1 and serum BR/BV can be used as biomarker for autoimmune diseases. While, the potential of serum ferritin as a biomarker in systemic lupus erythematosus (SLE) or rheumatoid arthritis (RA) diagnosis process needs further verification. Serum ferritin levels are positively correlated with disease activity index in SLE and RA patients. In addition, the serum levels in patients with SLE and RA are lower than healthy controls and were inversely correlated with disease severity. In inflammatory bowel disease (IBD) and experimental autoimmune encephalomyelitis (EAE) diseases, the level of HO-1 in lesions rises along the disease course, and the induction of HO-1 could improve diseases severity.

The heme molecule provides a multitude of crucial biological functions, including oxygen transportation, signal transduction, peroxide metabolism and mitochondrial bioenergetics in the form of various apo-heme proteins like hemoglobin, myoglobin, and cytochromes (Ponka, 1999). Therefore, it is important for life and has attracted numerous researchers for decades. In 1951, Shemin and colleagues unraveled the set of enzymes involved in the synthesis of heme for the first time (Shemin and Wittenberg, 1951; Shemin, 1970; Shemin, 1989). Later in 1968, heme oxygenase (HO), the heme- degrading enzyme, was discovered (Tenhunen et al., 1968). Then the complete catabolic pathway of heme has been deciphered: HO catabolizes the first and rate-limiting step in the degradation of free heme into three products: carbon monoxide (CO), ferrous iron (which is quickly sequestered by ferritin), and biliverdin (BV) (which is converted to bilirubin (BR) by the enzyme biliverdin reductase (BVR)) (Wagener et al., 2003) (**Figure 1**).

Over the years, heme has been proven to play a role in regulating a wide spectrum of gene expression, cell differentiation, proliferation and immune stimulation, in addition to its function as a prosthetic moiety in heme proteins (Poulos, 2014; Fujiwara and Harigae, 2015; Ponka et al., 2017). Besides, HO (especially HO-1) has rapidly gained interest from a group of immunologists since this enzyme shows powerful anti-inflammatory and anti-oxidant properties (Loboda et al., 2016; Riquelme et al., 2016; Ryter and Choi, 2016; Vijayan et al., 2018). Notably, the heme-degradation products, CO, iron-induced ferritin and bilirubin, may also contribute to the beneficial effects of HO-1 activation (Lee et al., 2016; Naito et al., 2016; Ryter and Choi, 2016; Gomperts et al., 2017; Wilson et al., 2017). Recently, overwhelming evidence indicates that the heme catabolic pathway is tightly involved in the physiological or pathological processes such as cytoprotection, oxidative stress, apoptosis and inflammatory injury (Maines, 1997; Onyiah et al., 2013; Zhang et al., 2019). Inflammatory disease like inflammatory bowel disease (IBD) and autoimmune diseases such as systemic lupus erythematosus (SLE), rheumatoid arthritis (RA), and multiple sclerosis (MS) are all associated with oxidative damage and inflammatory injury (Zhang and Li, 2014; Kaul et al., 2016; Smolen et al., 2016; Correale et al., 2017). It is therefore of utmost importance to better understand the role of heme catabolic pathway molecules in inflammatory and immune disorders and to develop the corresponding therapeutic strategies.

## Heme

Heme is an important iron-containing porphyrin molecule expressed ubiquitously in organisms. It is essential for several fundamental activities since it comprises the prosthetic moiety of diverse hemoproteins (Wagener et al., 2003), which are crucial for multiple biological processes including reversible oxygen binding and transport, mitochondrial electron transfer and oxidative reactions (Ryter and Tyrrell, 2000). In various pathologies including hemolytic diseases (such as sickle-cell disease, malaria, and β-thalassemia), rhabdomyolysis and subarachnoid hemorrhage, large quantities of hemoproteins are released into plasma (Kumar and Bandyopadhyay, 2005; Schaer et al., 2013). And then hemoproteins are oxidized and release the heme moiety, forming high levels of free heme and exerting pro-oxidant, pro-inflammatory and proliferative effects (Schaer et al., 2013). Besides, heme is also involved in the pathogenesis of sepsis, renal injuries and atherosclerosis (Luo et al., 2003; Morita, 2005; Mehta and Reddy, 2015; Deuel et al., 2016). To date, the direct pathological effects of heme have not been mentioned in autoimmune diseases.

**Figure 2:**
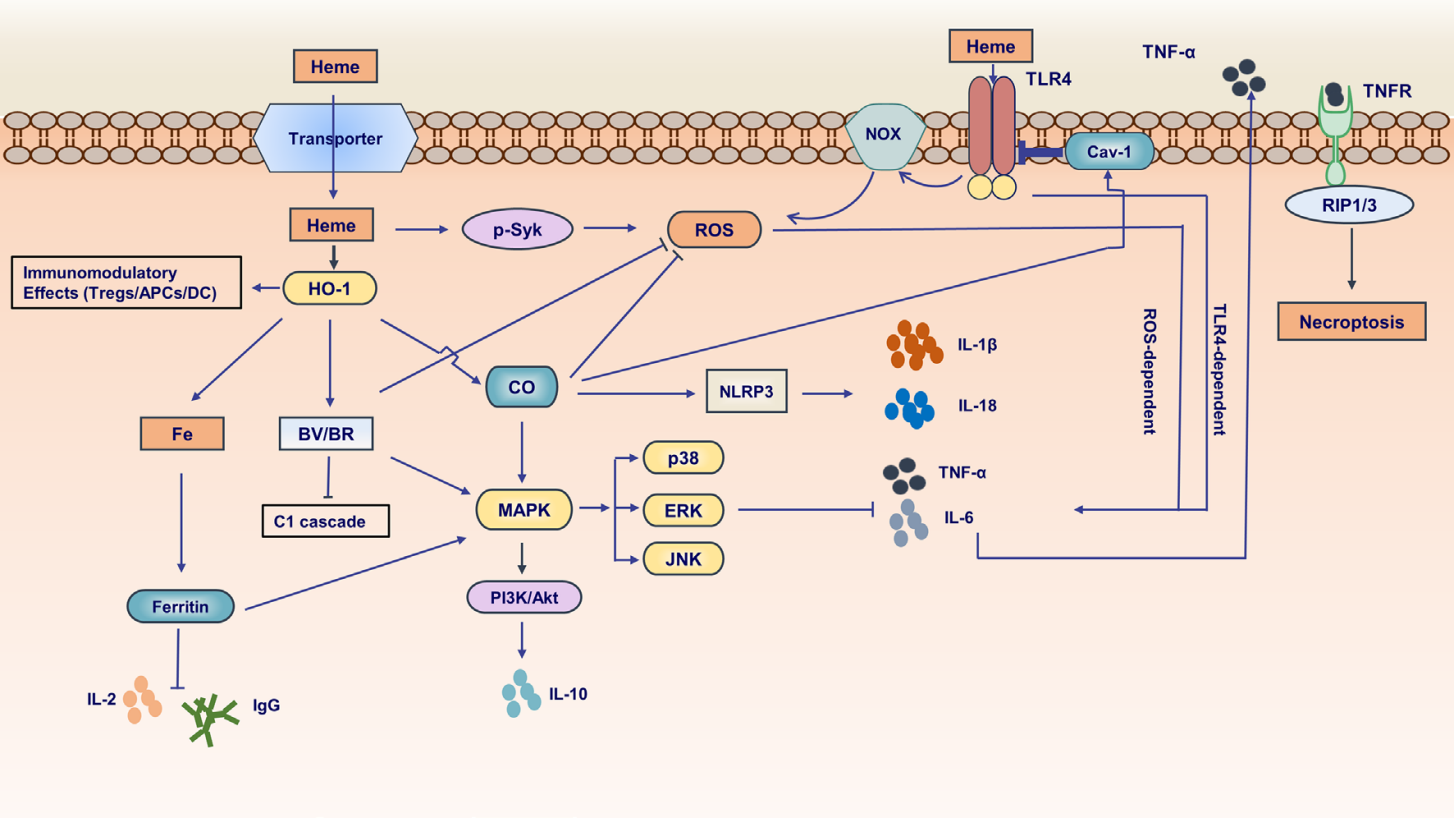
Pivotal functions of the molecules in heme metabolic pathway in inflammation. Free heme is transported by an unknown transporter into intracellular side. Heme can activate spleen tyrosine kinase (Syk) and subsequently induce reactive oxygen species (ROS) generation. Furthermore, heme can activate Toll-like receptor4 (TLR4) inducing ROS production dependent on NADPH oxidase and promoting proinflammatory cytokines [e.g., tumor necrosis factor-α (TNF-α)] generation. HO-1 have immunomodulatory effects in dendritic cells (DCs), antigen-presenting cells (APCs) and regulatory T cells. In heme metabolic pathway, the end product ferrous iron (Fe^2+^) is proinflammatory and could be sequestered by iron storage protein ferritin. The ferritin inhibits interleukin-2 (IL-2) and IgG production. In addition, it is involved in mitogen-activated protein kinase (MAPK) signaling pathway. CO downregulates the production of proinflammatory cytokines (e.g., TNF-α, IL-6) and upregulates the anti-proinflammatory cytokines (e.g., IL-10) relating to MAPK signaling pathway. CO inhibits ROS generation and regulates inflammasome activation followed by impacting IL-1β maturation and secretion. Of note, CO could augment the interaction between caveolin-1 (cav-1) and TLR4 suppressing TLR4-mediated signaling. BV/BR exerts anti-inflammatory and anti-oxidant effects about innate and adaptive immunity. The production of pro-inflammatory cytokine TNF-α can be secreted into extracellular side. TNF-α binds to its receptor inducing necrosis mediated by receptor-interacting protein (RIP1/3).

Free heme causes inflammation mainly through two mechanisms (Dutra and Bozza, 2014): 1) intercalating in membrane and altering cellular structures on account of the lipophilic property of heme (Balla et al., 1991; Beri and Chandra, 1993; Ryter and Tyrrell, 2000); 2) activating immune responses and inflammatory reactions which act as the pro-oxidant in endothelial cells, neutrophils, and macrophages (Graca-Souza et al., 2002; Fernandez et al., 2010; Mocsai, 2013; Belcher et al., 2014; Chen et al., 2014; Vinchi et al., 2016; Petrillo et al., 2018). Exposure of heme to endothelial cells stimulated the expression of adhesion molecules, such as ICAM-1 (intercellular adhesion molecule 1) and VCAM-1 (vascular cell adhesion molecule 1), probably through heme-mediated reactive oxygen species (ROS) generation and transcription factors NF-κB signaling pathway activation (Wagener et al., 1997; Wagener et al., 2001; Belcher et al., 2014). Adhesion molecules make leukocytes attach firmly to the endothelium and migrate to tissue parenchyma, behaving like one of the main characteristics of inflammation. A study showed that heme activates neutrophils through protein kinase C (PKC) activation and NADPH oxidase-dependent ROS generation, inducing the expression of adhesion molecules, which are indispensable for neutrophil migration (Graca-Souza et al., 2002). Other studies suggested that heme induces neutrophil migration caused by activating of G-protein-coupled receptor (Porto et al., 2007) or by mediating macrophage-derived leukotriene B4 (LTB4) production (Monteiro et al., 2011). Especially, heme could delay neutrophils apoptosis *in vitro* through the phosphoinositide 3-kinase (PI3K) and NF-κB pathways, increasing their longevity and upregulating their harmful stimuli from these non-apoptosis cells (Arruda et al., 2004). Heme also amplifies the innate immune response to microbial molecules through spleen tyrosine kinase (Syk)-dependent ROS generation (Fernandez et al., 2010) (**Figure 2**). Of note, heme induces TLR4-triggered tumor necrosis factor (TNF)-α production and ROS generation in macrophages (Figueiredo et al., 2007). In addition, free heme could induce vascular occlusion and acute lung injury in a sickle-cell disease mouse model by activating TLR4 signaling (Ghosh et al., 2013; Belcher et al., 2014). As a damage-associated molecular pattern (DAMP), heme induces autocrine TNF-α and ROS production in macrophages, leading to programmed necrosis dependent on the receptor-interacting protein (RIP) 1/3 (Fortes et al., 2012). More recently, it has also been found that heme could activate the nucleotide-binding domain and leucine-rich repeat pyrin 3 containing (NLRP3) inflammasome in macrophages and mouse models of hemolysis (Dutra et al., 2014). The above results showed that excessive free heme might be an important pathogenic factor in inflammatory disorders.

**Figure 3:**
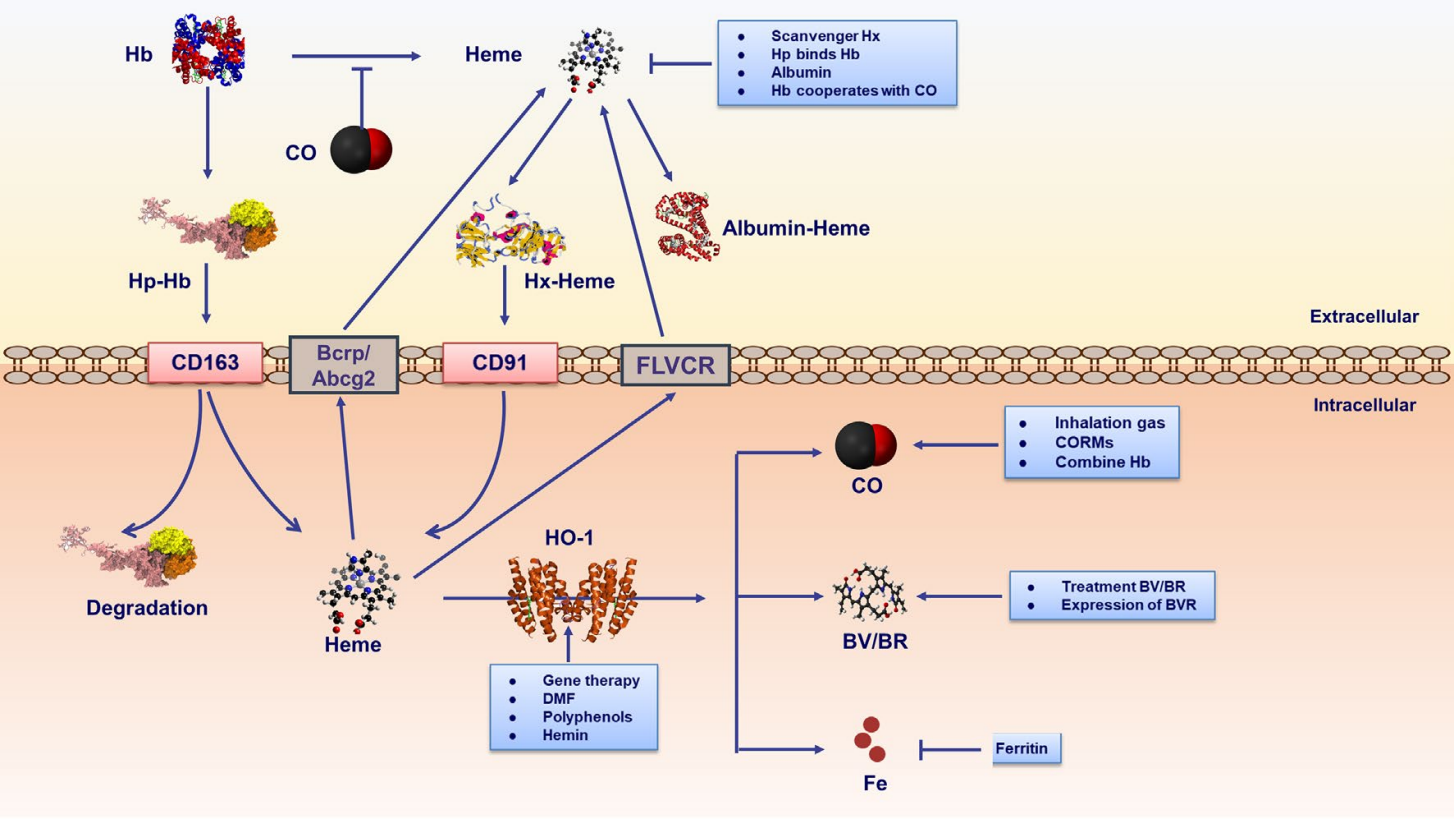
Therapeutic strategies are involved in heme metabolic pathway. strategies include extracellular and intracellular ways. Extracellular mechanisms mainly conclude scavenger proteins haptoglobin (Hp)/hemopexin (Hx) binding hemoglobin (Hb)/free heme respectively. Furthermore, albumin binds free heme inhibiting its proinflammatory effects. Hp-Hb complex binds to CD163 receptor expressed on the cell membrane, leading to endocytosis and degradation of the Hb and Hp complexes. It is noteworthy that CO binding Fe^2+^ in hemoproteins could prevent heme releasing. Heme-Hx complex is recognized by cell receptor CD91, delivering heme for catabolism by HO-1. The intracellular heme is degraded by HO-1. In addition, cellular heme levels could be excluded by heme efflux transporters FLVCR and Bcrp/Abcg2. Gene therapy and HO-1 inducers could regulate HO-1. HO-1 inducers contain natural (polyphenols) and synthetic (DMF) compounds. In addition, as the substrate of HO-1, hemin is a natural HO-1 inducer existing in human body. The labile ferrous iron released from heme catabolism is subsequently sequestered by ferritin, thus conferring cyto-protection against heme-Fe^2+^. The end products of HO-1 activity, namely BV/BR, and CO act as pharmacologic molecules. CO could be elevated by inhalation or through the use of CO-releasing molecules (CORMs). Additional therapy involving CO includes CO-binding hemoglobins. BV/BR treatment and BVR expression is also proposed as intervention strategies.

There are three physiological approaches to regulating heme homeostasis and its potential toxicity (**Figure 3**). First, the proteins haptoglobin and hemopexin scavenge extracellular hemoglobin and free heme in plasma, respectively. Second, the intracellular heme is degraded by HO-1 (details in the following parts). A third regulator of cellular heme levels could be heme efflux transporters: FLVCR (feline leukemic virus receptor) and Bcrp/Abcg2 (breast cancer resistance protein/ATP-binding cassette g2).

Under pathological conditions such as hemolysis and tissue injury, large amounts of hemoglobin (Hb), myoglobin and other hemoproteins are unleashed into the plasma from damaged red blood cells (RBCs) (Reeder, 2010; Schaer et al., 2013). The scavenger protein haptoglobin (Hp) can rapidly bind with cell-free Hb and neutralize its pro-oxidative effects (Schaer et al., 2013). Once the binding capacity of Hp saturates, free Hb is rapidly oxidized and releases prosthetic heme groups (Bunn and Jandl, 1968; Hebbel et al., 1988; Balla et al., 1993; Ferreira et al., 2008). Similarly, free cytotoxic heme can also be accumulated by the oxidation of other hemoproteins (such as myoglobin) (Nath et al., 1992) and can be combined by heme scavenger proteins (hemopexin, albumin, and α-micro-globulin) within their binding capacity (Muller-Eberhard and Cleve, 1963; Muller-Eberhard, 1988; Miller and Shaklai, 1999; Allhorn et al., 2002; Tolosano and Altruda, 2002). Hb-Hp complex binds to CD163 expressed on macrophages and hepatocytes, and then the complex degrades in the cytoplasm (Larsen et al., 2012). Hp combined with Hb regulates the pathogenesis of immune-related diseases (Quaye, 2008). It is noteworthy that CO binding Fe^2+^ in hemoproteins could prevent heme releasing, suppressing the disease progression of malaria and other immune-related diseases (Pamplona et al., 2007; Ferreira et al., 2008; Gozzelino et al., 2010). Hemopexin (Hx), an acute phase protein in plasma, binds free heme with the high affinity (KD10^-14^) (Muller-Eberhard and Cleve, 1963; Muller-Eberhard, 1988; Tolosano and Altruda, 2002) *via* its characteristic heme-binding pocket (Paoli et al., 1999). The heme-hx complex is recognized by the macrophage receptor CD91 (Hvidberg et al., 2005) and further heme is degraded by the effect of HO enzymes. Hx is not degraded by macrophages, being transported back into the plasma for the circulation (Hvidberg et al., 2005). *Hx* and/or *Hp* gene knockout mice develop a normal phenotype in non-challenged conditions while exhibiting severe renal and hepatic damages when subjected to experimental hemolysis (Tolosano et al., 1999; Tolosano et al., 2002; Vinchi et al., 2008). Furthermore, *Hx* deficiency is involved in inflammatory diseases, such as septic shock and experimental autoimmune encephalomyelitis (EAE) (Lin et al., 2015; Mehta and Reddy, 2015). In lipopolysaccharide (LPS)-triggered macrophages, Hx down-regulates pro-inflammatory cytokines [such as TNF, interleukin-6 (IL-6) and IL-1β] production and acts as a negative regulator of Th17 response in EAE (Rolla et al., 2013). In a mouse model of sickle cell disease, Hx therapy (4 mg Hx intraperitoneally once a week, for 3 weeks) reverts heme-induced switching of macrophages from proinflammatory phenotype M1 following the decreasing of M1marker (CD86, iNOS, and MHC II) (Vinchi et al., 2016). These results suggest that Hx and Hp are part of systemic protective mechanisms against the free heme and may act as potential approaches for the treatment of immune and inflammatory disorders. In addition to Hx, albumin binds heme with a lower affinity than Hx (KD10^-8^) (Little and Neilands, 1960), while exhibiting higher concentration in plasma than Hx (Adams and Berman, 1980). Children with severe malaria (Maitland et al., 2005; Akech et al., 2006) and sepsis (Delaney et al., 2011) have significantly improved survival after the administration of albumin in clinical trials. However, whether the therapeutic effect of albumin is due to its heme scavenging capacity remains unclear and further studies should be established.

The intracellular localization and concentrations of heme are also tightly regulated. FLVCR and Abcg2 are identified as heme/porphyrin transport proteins localized on the plasma membrane, which move heme/porphyrins from the intracellular to the extracellular environment and protect cells from heme overloading. As we gain additional knowledge, therapeutic manipulation of the expression of these transporters could provide an alternative way of treating heme-related pathologic conditions (Krishnamurthy et al., 2007; Larsen et al., 2012).

## Heme Oxygenase: The Heme-Degrading Enzyme

Heme oxygenase (HO) is the primary and rate-limiting enzyme in the heme catabolic pathway and plays a pivotal role in protecting cells from heme-induced oxidative stimuli. It has three distinct isoforms (HO-1, HO-2, HO-3). HO-1 is an inducible enzyme, highly expressed by several stimuli like its substrate heme, heat shock, heavy metal irons, oxidative stress, inflammatory cytokines, and LPS. Whereas, HO-2 is constitutively expressed in most cells and takes part in regulating physiological processes (Maines, 1988). HO-3 has poor heme-degrading capacity and the studies about HO-3 are still rather limited (Wagener et al., 2003). In recent years, HO-1 has been considered as an especially charming molecule for the prevention and management of immune-mediated injuries and diseases. In mammals, HO-1 is encoded by the *HMOX1* gene. Studies find that the *HMOX1* genes are highly transcriptionally regulated by injurious stimuli. HO-1 deficiency presents a chronic inflammation state featured by an increasing peripheral blood lymphocyte counts and accumulation of monocyte/macrophage in the spleen (Poss and Tonegawa, 1997). It has been shown that HO-1 induction by a retroviral vector could suppress TNF-induced cell death (Kushida et al., 2002). In ovalbumin-induced allergic asthma mice, HO-1 inhibits basophil maturation and activation and promotes basophil apoptosis (Zhong et al., 2016). These results show that HO-1 has an anti-inflammatory effect.

In recent years, an increasing number of experiments have proved the protective effects of HO-1 in autoimmune diseases. Patients with SLE show decreased expression of HO-1 in circulating monocytes, raising the possibility of a connection between myeloid cell HO-1 expression and lupus nephritis (Herrada et al., 2012). Besides, Mackern-Oberti et al. showed that HO-1 mRNA transcription is reduced in spleen inflammatory cells of FcγRIIb^−/−^ mice, a model for SLE. And HO-1 induction or CO treatment could ameliorate the proteinuria and renal inflammation in this model, which provides evidence for an anti-inflammatory and reno-protective role for HO-1 (Mackern-Oberti et al., 2013). Takeda and co-workers reported that induction of HO-1 with hemin mitigates lupus nephritis in MRL/*lpr* mice by reducing local inducible nitric oxide synthase (iNOS) expression, decreasing the levels of anti-dsDNA antibody and IFN-γ in serum (Takeda et al., 2004). These experiments manifest that in SLE disease, HO-1 may act as a useful marker and HO-1 induction might be a novel therapeutic strategy.

The protective role of HO-1 in autoimmune diseases could be further corroborated in rheumatoid arthritis. In the collagen-induced arthritis (CIA) model, HO-1 is remarkably induced in inflamed tissues (Devesa et al., 2005b). HO-1 is also highly expressed in synovial tissues (Kobayashi et al., 2006) and synovial fluid of rheumatoid arthritis patients (Kitamura et al., 2011). Besides, the induction of HO-1 reduces TNF-α and suppresses LPS-induced production of IL-6 and IL-8 in synovial cell lines (Kobayashi et al., 2006). Moreover, IL-6 and matrix metalloproteinase-3 (MMP-3), which are markers of joint destruction and synovial tissue proliferation (Kitamura et al., 2011), significantly increase in HO-1 deficient arthritic mice (Brines et al., 2012). Bone morphogenetic protein (BMP) is also recognized as a marker of bone metabolism and HO-1 level is correlated to BMP in RA patients (Yuan et al., 2016). These data underscore that HO-1 acts as a potential biomarker and therapy for RA.

The role of HO-1 was also reported in other rheumatic conditions. HO-1 activation significantly alleviates imiquimod-induced psoriasis by downregulating IL-6/IL-22-induced Stat3 activation (Zhang et al., 2016). Additionally, HO-1 is induced to downregulate retinoic acid-related orphan receptor γt expression and IL-17A levels, while promoting the expression of Treg-related forkhead box p3 (Foxp3) and IL-10 level in dextran sulfate sodium (DSS)-induced acute murine colitis (Zhong et al., 2010; Zhang et al., 2014). In 2,4,6-trinitrobenzene sulfonic acid (TNBS)-induced colitis model, hemin-treated mice present a decrease in fecal hemoglobin, alkaline phosphatase (ALP), and proinflammatory cytokine concentrations (TNF-α and IL-1β) (Mateus et al., 2018). In EAE, the level of HO-1 in lesions rises along the disease course (Liu et al., 2001; Wang et al., 2017), indicating ongoing oxidative stress and endogenous activation of antioxidant defense. It was also demonstrated that hemin or Co-PPIX, as an inducer of HO-1, inhibits EAE effectively. In contrast, tin mesoporphyrin, an inhibitor of HO-1 activity, markedly exacerbated EAE (Chora et al., 2007). Besides, HO-1 expression decreased in immune cells from systemic sclerosis (SSc) patients, whereas Co-PPIX treatment could restore HO-1 levels in DCs and normalize the increased TLR response observed in SSc (van Bon et al., 2016). Remarkably, some studies indicated that HO-1 is not always related to beneficial effects. For example, HO-1 inhibition exerts antioxidant effects in EAE model (Chakrabarty et al., 2003) and rat adjuvant arthritis (Devesa et al., 2005a).

The above data suggest that upregulating HO-1 may act as a potential therapy strategy in treating immune diseases. In brief, there are mainly two ways of improving HO-1 level: HO-1 inducers and gene therapy of *HMOX1* (**Figure 3**). HO-1 inducers include natural and synthetic compounds. As the substrate for HO-1, heme is a natural HO-1 inducer that exists in the human body. Therefore, exogenous administration of hemin is a widely used method to explore the role of HO-1 in immune diseases (Zhang et al., 2014; Zhong et al., 2016). In addition, there are another two kinds of HO-inducing agents including plant-derived polyphenols and pharmaceutical compounds (Ryter and Choi, 2016). HO-1 inducers of plant-derived compounds contain curcumin, caffeic acid, carnosol and others (Ryter and Choi, 2016). However, there is no study on human immune disorders, and further studies are essential to identify safety and efficacy based on these compounds. The representative pharmaceutical compound of the HO-1 inducer is dimethyl fumarate (DMF), showing therapeutic effects on treating multiple sclerosis (MS) (Nicholas et al., 2014; Dubey et al., 2015). The second way to improve HO-1 level is gene therapy is as follows. Some lines of studies have demonstrated that HO-1 could be upregulated *via* adenovirus- and retroviral-mediated gene transfer *in vivo*, exerting its anti-inflammatory effects in inflammation and immune disorders (Otterbein et al., 1999; Chauveau et al., 2002; Liu et al., 2006; Abraham et al., 2007; Cao et al., 2011; Petersen et al., 2011; Cao et al., 2012). In addition, in bone-derived macrophages, transduction of the HO-1 gene rapidly reduces TNF-α and increases IL-10 cytokines after LPS stimulation (Ferenbach et al., 2010). However, further studies considering the safety and efficacy of HO-1 gene application in immune and inflammatory disorders remain to be established.

## Ferrous Iron

Ferrous iron is a pro-oxidative and pro-inflammatory metabolite in the heme catabolic pathway. It promotes the formation of free radicals through the Fenton reaction, which catalyzes Fe and H_2_O_2_ into hydroxyl radicals (Rodopulo, 1951).

Excessive ferrous iron is effectively captured by ferritin, which is a ubiquitously existing intracellular iron storage protein. Ferritin is composed of ferritin heavy (H) chain and light (L) chain (Torti and Torti, 2002). Ferritin H has ferroxidase activity and converts the ferrous (Fe^2+^) iron into ferric (Fe^3+^) form (Honarmand Ebrahimi et al., 2015). The light chain is involved in iron nucleation and transfers electrons across the protein cage (Carmona et al., 2014). In most tissues, ferritin is a cytosolic protein regulating iron deficiency and overload. Small amounts of ferritin are secreted into the serum as an indirect marker of iron content in the body (Wang et al., 2010).

The expression of ferritin is delicately regulated in transcriptional, translational, and even post-transcriptional levels (Torti and Torti, 2002; Zandman-Goddard and Shoenfeld, 2007). When the iron level is low, ferritin synthesis decreases and vice versa. Ferritin synthesis is also regulated by cytokines. For example, both TNFα and interferon γ could induce the ferritin H mRNA expression in the U937 macrophage cell. IL-1β also affects ferritin accumulation post-transcriptionally in human astrocytoma cells and, thus, reduces the labile iron pool (You and Wang, 2005). Other stimuli, such as reactive oxygen and nitrogen species and hypoxia, can also alter iron regulatory proteins binding activities and content, and consequently affect ferritin translation (Recalcati et al., 2008).

In 1981, Broxmeyer et al. first found that ferritin represses the production of granulocytes and macrophages, and subsequently extended this by showing that H-ferritin is involved in the negative regulation of human and murine hematopoiesis. H-ferritin can also suppress the proliferation of T cells in response to mitogens and impair the maturation of B cells (Morikawa et al., 1995; Zandman-Goddard and Shoenfeld, 2007). In LPS-induced Raw264.7 cells, overexpression of ferritin L chain significantly decreases pro-inflammatory cytokines (TNF-α, IL-1β) and NO production and inhibits MAPKs and NF-κB pathways activation (Fan et al., 2014).

In addition, the ferritin H chain mediates the protective effect of HO-1 against oxidative stress. HO-1 RNAi makes the cells more susceptible to hydrogen peroxide, which could be rescued by ferritin H chain expression (Cheng et al., 2015). In heme-exposed endothelial cells, ferritin could be induced by excess iron and protects cells from oxidative damage.

Evidence demonstrated that the ferritin H chain also affects chemokine receptor signaling and receptor-mediated cell migration. It has been shown that ferritin H chain is a negative regulator of CXC chemokine receptor 4 (CXCR4) and overexpression of ferritin H chain leads to the inhibition of MAPK signaling, a kinase in regulating migration, differentiation, and proliferation (Li et al., 2006).

Several studies have reported the nuclear localization of ferritin, encouraging a new perspective on ferritin in the role of DNA protection and transcriptional regulation (Alkhateeb and Connor, 2010). Studies on the corneal epithelium demonstrated that ultraviolet radiation and H_2_O_2_ inducing DNA double-strand breaks, thymine dimers, and ROS generation are rescued by nuclear ferritin (Liu et al., 1996; Shimmura et al., 1996; Cai et al., 1998; Linsenmayer et al., 2005; Cai et al., 2008). A recent study found that in epithelial cells, localization of ferritin to nucleus, decreased JNK pathway activity and inhibition of ferritin synthesis leads to increased JNK phosphorylation (Kubilus et al., 2016). Ferritin H chain having ferroxidase activity bound the β-globin gene promoter (Surguladze et al., 2004). These results reveal that the translocation of ferritin into the nucleus might exert a protective effect on DNA when cells meet inflammation or oxidative damage.

It was shown that the levels of ferritin as an acute-phase effector change in various immune disorders. Studies on different ethnic groups [Japanese (Nishiya and Hashimoto, 1997), Korean (Lim et al., 2001), Turkish (Beyan et al., 2003), and American (Vanarsa et al., 2012)] have shown that serum ferritin levels are positively correlated with disease activity index of active SLE patients compared with inactive cases. Especially, the increasing levels of ferritin correlate with renal dysfunction in SLE (Tripathy et al., 2015). In line with previous reports in Korean (Lim et al., 2001) and the Japanese population (Nishiya and Hashimoto, 1997). An Indian cohort survey (Tripathy et al., 2015) found serum ferritin level inversely correlates with complement components C3, C4 and positively with anti-dsDNA levels (Lim et al., 2001). Based on these results, ferritin may act as a biomarker evaluating disease activity degree in SLE patients.

In addition, elevated levels of ferritin are detected in both synovial fluid (Blake and Bacon, 1981; Milman et al., 1985) and synovial cells (Muirden, 1966) in RA patients. Compared with OA patients, high serum ferritin levels are found in RA patients (Ota and Katsuki, 1998). However, in another study, serum ferritin levels are not significantly changed in RA patients and normal subjects (Rothwell and Davis, 1981; Kumon et al., 1999). Furthermore, the difference between ferritin level and RA disease activity may be mediated by the following factors: 1) active or inactive phase of RA; 2) The number of RA patients enrolled in these studies; 3) The high rate of iron deficiency in RA (Seyhan et al., 2014); 4) The different evaluation criteria for RA disease activity. To conclude, more research is indispensable for identifying the relationship between ferritin and RA.

## Carbon Monoxide (CO)

CO is produced during the catabolism of free heme. It is widely considered as a toxic gas, since it binds to hemoglobin better than oxygen, interfering with the oxygen-carrying capacity of the blood, leading to tissue hypoxia (Ryter and Otterbein, 2004; Ryter et al., 2018). However, a large amount of evidence proved that CO in a non-toxic concentration exerts its physiological and cytoprotective effects in response to cellular stress in the pathological processes of inflammation and immune disorders (**Figure 2**).

It is worth mentioning that CO could bind Fe^2+^ in heme groups and prevent the oxidation of hemoproteins (Balla et al., 1993). In earlier research, Otterbein and colleagues demonstrated that CO inhalation exerts anti-inflammatory effects in macrophages challenged with LPS (Otterbein et al., 2000). CO inhibits the secretion of pro-inflammatory cytokines (TNF-α, IL-1β) and macrophage inflammatory protein-1 through the p38 MAPK pathway, whereas, it increases the expression of the anti-inflammatory cytokine IL-10 (Otterbein et al., 2000). Consistent with this notion, CO depresses T cell proliferation and IL-2 expression *via* inhibiting ERK pathway (Pae et al., 2004) and decreases IL-6 production *in vivo* through JNK pathway in a mouse model of sepsis (Morse et al., 2003). In human colonic epithelial cells, CO inhibits iNOS expression and IL-6 secretion by regulating the NF-κB and MAPK pathways (Megias et al., 2007). In addition, CO inhibits the transport of TLR to lipid rafts by suppressing the production of NADPH oxidase-dependent ROS (Nakahira et al., 2006), affecting the TLR4 signaling pathway. Caveolin-1 (cav-1), the basic structural protein of plasmalemmal caveolae, exerts anti-inflammatory effects through preventing TLR4 association with MyD88 and TRIF and downregulating activation of the NF-κB pathway. Remarkably, CO could augment cav-1/TLR4 interaction (Wang et al., 2009). In LPS-stimulated cystic fibrosis macrophages, TLR4 signaling is activated and the stress-induced expression of HO-1 is recruited to the cell surface with cav-1, suppressing TLR4-mediated signaling by CO generation (Zhang et al., 2013).

What’s more, current research reveals the potential regulatory effect of CO in the inflammasome system. Under stress conditions, inflammasome assembly promotes the auto-cleavage of caspase-1 and then leads to the maturation and secretion of pro-inflammatory cytokines IL-1β and IL-18. NLRP3 inflammasome, which is composed of NLRP3, the adaptor protein ASC and caspase-1, is the pivotal research object in immune responses (Latz et al., 2013; de Zoete et al., 2014). Studies demonstrated that CO-releasing molecule 2 (CORM-2) inhibits caspase-1 activation and IL-1β secretion in response to the endoplasmic reticulum (ER)-stress induced inflammation (Kim et al., 2014). In addition, CO inhibits mitochondrial ROS generation and decreases mitochondrial membrane potential induced by LPS and ATP in macrophages (Jung et al., 2015). By contrast, it was shown that macrophage generated CO promotes ATP production and release by bacteria, which activates NLRP3 inflammasome by activating the purinergic receptor (P2X7R) (Wegiel et al., 2014). Therefore, further research is needed to illustrate the precise mechanisms. The effect of CO on the inflammasome may depend on distinct pathogens, pathogen-associated molecular patterns (PAMPs) or DAMP. In summary, CO might be a potent regulator of the inflammasome.

The therapeutic effects of CO on autoimmune diseases were also confirmed. As mentioned above, a study found that administration of CO could decline the expansion of CD11b^+^ cells, prevent the reduction of regulatory T CD4^+^ Foxp3^+^ cells, and lessen anti-histone antibodies in an FcγRIIb receptor knock-out lupus-prone mice. In addition, animals treated by CO manifest alleviated kidney damages compared with untreated mice (Mackern-Oberti et al., 2013). Similarly, in MRL/*lpr* lupus mice model, CO inhalation significantly decreases the proportion of activated B220^+^CD4^-^CD8^-^T cells in kidney, and levels of anti-nuclear antibodies (ANA) and anti-histone antibodies (Mackern-Oberti et al., 2015). Therefore, the inflammatory environment mediated by an elevated number of monocytes, and self-antigen-specific T cells can be controlled by the expansion regulatory/anti-inflammatory T cells after administration of CO. Inhalation of CO also ameliorates collagen-induced arthritis in mice and regulates the articular expression of IL-1beta and MCP-1 (Takagi et al., 2009). In other research, similar results were obtained indicating that the administration of CO decreases serum anti-collagen II antibodies, ameliorates disease activity and displays lower inflammation and cartilage damage in CIA-induced mice arthritis (Bonelli et al., 2012). In addition, inhalation of CO significantly inhibits inflammatory responses in LPS-induced human rheumatoid arthritis synovial fibroblasts (RASFs) by downregulating the expression of adhesion molecule VCAM-1 and leukocyte infiltration (Chi et al., 2014). In EAE, mice treated with CO-releasing molecule [(CORM)-A1] shows reduced cumulative score, shorter duration and decreased cumulative incidence of the disease as well as milder inflammatory infiltrations in the spinal cords (Fagone et al., 2011). These findings suggest that CO exposure is a potential strategy for autoimmune diseases.

From the above, the end product CO exhibits a promising strategy in inflammatory and immune disorders. There are two ways to increase the levels of CO: inhalation CO gas and application of chemical CORMs (**Figure 3**). CO is colorless and odorless gas, which is easy to get in large quantities. Many lines of studies have demonstrated that inhalation of CO gas in a non-toxic concentration exerts anti-inflammatory and cytoprotective actions through different pathways (Otterbein et al., 2000; Morse et al., 2003; Pae et al., 2004; Nakahira et al., 2006; Megias et al., 2007; Wang et al., 2009; Zhang et al., 2013; Kim et al., 2014; Jung et al., 2015). As described, evidence also showed that CO inhalation is a potential strategy in SLE and RA treatment (Tayem et al., 2006; Takagi et al., 2009; Mackern-Oberti et al., 2013; Mackern-Oberti et al., 2015). Besides, adoption of CORMs is another option. To date, several CORMs have been applied including: water-soluble CORM-3 [Ru (CO3)-glycinate] and CORM-A1 (sodium boranocarbonates) (Clark et al., 2003; Motterlini et al., 2005; Johnson et al., 2007) and dimethyl sulfoxide-soluble CORM-2 {[Ru (CO3) Cl2] 2} (Motterlini et al., 2002). CO is generated by these compounds by various stimuli such as changes in pH, redox reactions and light activation (Motterlini et al., 2002; Motterlini et al., 2005). The different CO-releasing rates in these compounds determine their pharmacological activities *in vitro* and *in vivo* (Clark et al., 2003; Motterlini et al., 2005). The half-life of CORM-3 is less than 1 min, whereas the half-life of CORM-A1 is 21 min, although they both are water-soluble compounds (Motterlini et al., 2003). These different chemical properties resolve their effects and CORM-3 is used in an acute condition while CORM-A1 in mild diseases such as vasodilatory and hypotension (Foresti et al., 2004). Some lines of evidence have shown that CORMs exert cytoprotective and anti-inflammatory functions. The inflammatory response and cartilage destruction are declined in CIA-induced RA mice following intraperitoneal injection (10 mg/kg/day) of CORM-3 (Ferrandiz et al., 2008; Maicas et al., 2010). In LPS-stimulated Raw 264.7 macrophages, CORM-2 reduces the production of pro-inflammatory cytokines and suppresses iNOS activity and NO production (Tsoyi et al., 2009). CORM-2 prolongs survival and reduces inflammation injury when lung and liver are attacked by LPS in mice (Sarady et al., 2004; Cepinskas et al., 2008). CORM-A1 liberates CO in a pH and temperature manner (Motterlini et al., 2005) and inhibits ROS generation and apoptosis induced by TNF-α in murine intestinal epithelial MODE-K cells (Babu et al., 2015). To conclude, CO gas and CORMs may act as a potential treatment strategy in immune disorders, and further studies are necessary to explain unclear mechanisms as well as their clinical use.

## Biliverdin/Bilirubin

Biliverdin (BV), a water-soluble molecule with tetrapyrrole structure, is produced in heme metabolism and is subsequently converted to bilirubin (BR) through biliverdin reductases (BVR) (Tenhunen et al., 1968; Maines, 2000). Accumulating evidence suggests that the concentration of BR varies widely as the host’s physiology changes and has different effects based on its concentration. When BR production is increased (such as due to excessive hemolysis) and/or glucuronidation is impaired, it accumulates in blood. Once the plasma albumin-binding capacity for BR declines, the free (unbound) BR accumulates and exerts a pathological role in some diseases or processes (Jangi et al., 2013). In the last few decades, the beneficial properties of BV, BR, and BVR have been demonstrated in biological activities, including antioxidant functions and immunomodulation (Wang et al., 2004; Hu et al., 2015; Lee et al., 2016).

BR plays a crucial part in innate immunity. It affects the immune system depending on complement cascade by interrupting binding of the C1 complex to antibodies (Basiglio et al., 2007). In addition, BV administration significantly inhibits LPS-induced complement receptor 5a (C5aR) expression *via* the mTOR pathway and reduces the generation of complement-associated pro-inflammatory cytokine TNF-α and IL-6 in primary and immortalized macrophage cell lines (Bisht et al., 2014). A study proved that BR (but not CO) administration dose-dependently interferes INF-γ-induced JAK/STAT-1 signal transduction pathway and suppresses MHC- II expression in murine endothelial cells (2F2B) (Wu et al., 2005). In an autoimmune encephalomyelitis SLJ/J mice model, exogenous bilirubin supplement also could down-regulate MHC-II expression in APCs and suppress the expression of CD28, B7-1, B7-2 costimulatory molecules in T cells (Liu et al., 2008). Additionally, BR alters the expression subsets of the Fc receptor on the macrophage surface and regulates the macrophage’s phagocytic and anti-presenting function (Vetvicka et al., 1985). BR also plays a pivotal role in neutrophils. Enhancement of BR made neutrophils lose their ability for phagocytosis (Thong et al., 1977), migration and responsibility of chemotactic signals (Miler et al., 1981; Svejcar et al., 1984). BR scavenges ROS produced by neutrophils, consequently impairs neutrophil bacterial killing ability in a dose-dependent manner (Arai et al., 2001). Moreover, BR could increase heme-dependent enzymes (NADPH oxidase-1 and COX-2) generation in neonatal neutrophils (Weinberger et al., 2013) and inhibit production of adhesion molecules (VCAM-1, ICAM-1 and E-selectin) induced by TNF-α (Mazzone et al., 2009). The above data demonstrate that physiologic serum concentration of BR has the capacity to modulate innate immunity by interfering with the complement system, regulating Fc receptors and MHC II expression and levels of adhesion molecules in immune cells.

Except for its role in innate immunity, BR can influence adaptive immune response. BR is shown to decrease IL-2 production and inhibit T cell proliferation induced by phytohemagglutinin A (PHA) (Haga et al., 1996). Other studies have found that BR acts as a significant immunomodulatory agent in EAE mice. It could inhibit T cell proliferation, promote apoptosis in reactive T cells and decrease the production of pro-inflammatory Th1 cytokines (IL-2, IFN-γ) in a dose-dependent manner. Interestingly, BR treatment cannot upregulate the production of anti-inflammatory Th2 cytokines (IL-4 and IL-10), suggesting that BR does not lead to a skewing of the immune response from a Th1 cell to Th2 cell response (Liu et al., 2008). In macrophages, BR treatment induces an increasing expression of PD-L1 and further coculturing these macrophages with splenocytes leads to expansion of Foxp3^+^ Treg cells (Adin et al., 2017).

BVR is an enzyme involved in converting BV to BR, exerting anti-inflammatory and antioxidant actions in some immune diseases. BVR acts as a transcriptional factor on HO-1regulation (Ahmad et al., 2002). The function of BV relies on the increasing level of HO-1 by BVR in the lung. Blockade of HO-1 activity by Sn-PP results in loss of BV inhibitory effects on LPS-induced lung injury (Sarady-Andrews et al., 2005). As described in another article, the treatment of BV may trigger a feedforward cycle. Upregulation of HO-1 increasing BVR activity leads to the generation of endogenous products, such as CO, ferritin, or more BV, which exert their functions in some processes (Sedlak and Snyder, 2004). Although it has been established that BV could inhibit NF-κB activity in response to TNF-α (Gibbs and Maines, 2007) or LPS (Wegiel et al., 2009), BVR itself may have an effect on NF-κB on account of its kinase and reductase activity. The S/T/Y (serine/threonine/tyrosine) kinase activity has been linked to the insulin receptor signaling cascade in both the MAPK and PI3K/Akt pathways (Salim et al., 2001; Maines, 2005). It has been proven that overexpression of BVR enhances both the basal and TNF-α mediated NF-κB activation, influencing iNOS gene expression in HEK293 cells (Gibbs and Maines, 2007). Whereas, BVR inhibits NF-κB activation in response to LPS in macrophages and the effects are amplified with BV (Wegiel et al., 2011). Furthermore, Tat-BLVRA protein is effective in inhibiting MAPKs and NF-κB activation in LPS-stimulated Raw 264.7 cells (Kim et al., 2015). These differences between macrophages and HEK293 cells may be due to the types of receptors (TLR/TNFR), which both participate in the downstream-NF-κB signaling. In addition, BVR expression increases in M2 macrophages, which are associated with increased anti-inflammatory cytokine IL-10 generation (Hu et al., 2015). Consistently, others also found that cell surface BVR mediates biliverdin-induced anti-inflammatory effects through enhanced expression of IL-10 (Wegiel et al., 2009). These reports suggested that BVR may regulate the progression of inflammation *via* the IL-10 pathway. IL-10 production is associated with PI3K/Akt pathways. The effects of BV on IL-10 expression are lost with blockade of Akt. It has been demonstrated that BVR is identified to bind with PI3K-p85α driving Akt signaling (Wegiel et al., 2009), protecting against hypoxia by activation of PI3K and Akt (Pachori et al., 2007; Zeng et al., 2008). Inhibition of surface BVR with RNAi attenuates BV-induced Akt signaling and IL-10 expressions (Wegiel et al., 2009). In addition, the effect of BVR is associated with its nuclear function. Binding of BVR to Ap-1 sites could activate HO-1 gene expression (Ahmad et al., 2002; Kravets et al., 2004) and inhibit TLR4 gene transcription (Wegiel et al., 2011). The study found that BV triggers phosphorylation of endothelial nitric oxide synthase (eNOS) through calmodulin-dependent kinase (CaMK) in macrophages, increasing NO generation. The generated NO, in turn, nitrosylates BVR, leading to nuclear translocation where BVR binds to the TLR4 promoter at the Ap-1 sites to block transcription. Stable knockdown of BVR in macrophages leads to elevated expression of the TLR4 and pro-inflammatory cytokine TNF-α (Wegiel et al., 2011).

There are several studies on BR level and autoimmune diseases activity. SLE patients without liver diseases had significantly lower serum BR levels than the healthy control population. These effects can be observed in both male and female patients (Vitek et al., 2010; Yang et al., 2012; dos Santos et al., 2013). Serum BR levels are reduced more in SLE patients than pleuritis/nephritis (Vitek et al., 2010), which is negatively correlated with disease activity, antinuclear and anti-DNA antibodies (Yang et al., 2012). The decreasing level of BR may be caused by the severe oxidative stress consuming BR in SLE (Vitek et al., 2010). Interestingly, serum total or indirect BR levels in patients with SLE positively relate with high-sensitive C-reactive protein (hs-CRP) (Yang et al., 2012), which is an acute-phase protein in response to inflammation and/or tissue damage. However, it was proven that CRP remains low even at peak of SLE disease and might be a potential protector in SLE (Becker et al., 1980; Gershov et al., 2000; Marnell et al., 2005). These results provide a simple and effective method for SLE strategy by BR treatment. A large epidemiologic study using the National Health and Nutrition Examination Survey (NHANES) database observes that serum BR concentration has been confirmed to be related to a decreased risk of RA. High BR level is protective against RA (Fischman et al., 2010). In a CIA-induced murine RA model, BV treatment revealed that cartilage degradation is inhibited (Bonelli et al., 2012). Other surveys showed that RA patients have lower serum BR concentration than controls (Juping et al., 2017; Peng et al., 2017) and osteoarthritis (OA) (Juping et al., 2017), especially in RA patients with high disease activity individuals. Additionally, in IBD disease, BR treatment could prevent DSS-induced murine colitis by inhibiting the migration of leukocytes across the vascular endothelium and suppressing iNOS expression (Zucker et al., 2015). Consistent with this result, another study also found that bilirubin could relieve colitis by protecting intestinal barrier function, suppressing inflammation *via* the TLR4 or NF-κB signaling pathway (Zheng et al., 2019). Therefore, BR treatment holds promise as a therapeutic strategy for immune diseases including lupus, RA and IBD.

## Conclusion and Perspectives

Overwhelming evidence suggests that the heme catabolic pathway plays a vital role in the immune system. Its products (CO, BV/BR) exert important cytoprotective and anti-inflammatory effects during oxidative stress, inflammation, and especially immune disorders such as SLE, RA and MS and so on. In particular, removing excess heme with exogenous Hp or Hx, increasing HO-1 expression through gene therapy or chemical tools, administration of CO gas or CORMs, treatment with BV/BR, ferritin induction alone or in combination, not only alleviate heme cytotoxic properties but also reduce inflammatory reactions in immune disorders. Those results might direct the development of new therapeutic approaches in clinic. However, the enhancement of HO-1, ferritin, CO and BV/BR and the downregulation of free heme should be maintained at acceptable, non-toxic levels. Also, it is hoped that targeting heme metabolic pathway that have been proposed as effective therapeutic approaches for diseases such as lupus and RA, but whose effectiveness is yet to be formally proved, should be re-examined in the future. Further biomarkers should be exploited to improve the accuracy of autoimmune diagnosis and treatment. HO-1 and serum BR/BV may act as potential biomarkers for SLE/RA diagnosis and therapies. However, whether serum ferritin can be used as a biomarker in the SLE or RA diagnosis process remains to be demonstrated. At present, the investigation of the heme catabolic pathway is still in its infancy. Profound and detailed efforts and studies should be established to better understand and utilize downstream products in the metabolic pathway in treating immune and inflammatory disorders.

## Author Contributions

WT and YW conceived the review, participated in its design and helped to draft the manuscript. WB performed the material collection, data analysis, and manuscript writing. WT and YW revised the manuscript. All authors read and approved the final manuscript.

## Funding

This work was supported by the grants of National Science Fair Committee (NSFC), China (No. 81673445, 81803543), “Personalized Medicines-Molecular Signature-based Drug Discovery and Development,” Strategic Priority Research Program of the Chinese Academy of Sciences, Grant No. DA12020107, National Science & Technology Major Project ‘‘New Drug Creation and Manufacturing Program,’’ China (No. 2017ZX09101002-002-010). Project supported by the Shanghai Committee of Science and Technology,China (Grant No. 16431900300)

## Conflict of Interest Statement

The authors declare that the research was conducted in the absence of any commercial or financial relationships that could be construed as a potential conflict of interest.
